# Conserved Genomic Terminals of SARS-CoV-2 as Coevolving Functional Elements and Potential Therapeutic Targets

**DOI:** 10.1128/mSphere.00754-20

**Published:** 2020-11-25

**Authors:** Agnes P. Chan, Yongwook Choi, Nicholas J. Schork

**Affiliations:** a The Translational Genomics Research Institute (TGen), Phoenix, Arizona, USA; b Department of Population Sciences, The City of Hope National Medical Center, Duarte, California, USA; c Department of Molecular and Cell Biology, The City of Hope National Medical Center, Duarte, California, USA; Icahn School of Medicine at Mount Sinai

**Keywords:** SARS-CoV-2, human microRNA, influenza A H1N1, virus 3′ untranslated region, COVID-19

## Abstract

Severe acute respiratory syndrome coronavirus 2 (SARS-CoV-2) has infected over 40 million people worldwide, with over 1 million deaths as of October 2020 and with multiple efforts in the development and testing of antiviral drugs and vaccines under way. In order to gain insights into SARS-CoV-2 evolution and drug targets, we investigated how and to what extent the SARS-CoV-2 genome sequence differs from those of other well-characterized human and animal coronavirus genomes, as well as how polymorphic SARS-CoV-2 genomes are generally. We ultimately sought to identify features in the SARS-CoV-2 genome that may contribute to its viral replication, host pathogenicity, and vulnerabilities. Our analyses suggest the presence of unique sequence signatures in the 3′ untranslated region (3′-UTR) of betacoronavirus lineage B, which phylogenetically encompasses SARS-CoV-2 and SARS-CoV as well as multiple groups of bat and animal coronaviruses. In addition, we identified genome-wide patterns of variation across different SARS-CoV-2 strains that likely reflect the effects of selection. Finally, we provide evidence for a possible host-microRNA-mediated interaction between the 3′-UTR and human microRNA hsa-miR-1307-3p based on the results of multiple computational target prediction analyses and an assessment of similar interactions involving the influenza A H1N1 virus. This interaction also suggests a possible survival mechanism, whereby a mutation in the SARS-CoV-2 3′-UTR leads to a weakened host immune response. The potential roles of host microRNAs in SARS-CoV-2 replication and infection and the exploitation of conserved features in the 3′-UTR as therapeutic targets warrant further investigation.

**IMPORTANCE** The coronavirus disease 2019 (COVID-19) outbreak is having a dramatic global effect on public health and the economy. As of October 2020, SARS-CoV-2 has been detected in over 189 countries, has infected over 40 million people, and is responsible for more than 1 million deaths. The genome of SARS-CoV-2 is small but complex, and its functions and interactions with human host factors are being studied extensively. The significance of our study is that, using extensive SARS-CoV-2 genome analysis techniques, we identified potential interacting human host microRNA targets that share similarity with those of influenza A virus H1N1. Our study results will allow the development of virus-host interaction models that will enhance our understanding of SARS-CoV-2 pathogenesis and motivate the exploitation of both the interacting viral and host factors as therapeutic targets.

## Abstract (precis)

The CoV disease 2019 (COVID-19) infectious disease outbreak is having a dramatic global effect on public health and the economy. As of October 2020, SARS-CoV-2 has been detected in over 189 countries, has infected over 40 million people, and is responsible for more than 1 million deaths.

## INTRODUCTION

The coronavirus (CoV) disease 2019 (COVID-19) outbreak is having a dramatic effect not only on public health but also on the global economy. The acute respiratory distress associated with severe acute respiratory syndrome CoV-2 (SARS-CoV-2), the pathogen responsible for COVID-19 illness, was first reported in 2019 (1, 2). As of October 2020, SARS-CoV-2 has been detected in over 189 countries, has infected over 40 million people, and has been responsible for more than 1 million deaths (3; Johns Hopkins Coronavirus Resource Center, https://coronavirus.jhu.edu/map.html). The genome of SARS-CoV-2 is small but complex, encoding structural proteins and regulatory elements whose functions and interactions with host factors have been studied extensively (4, 5). However, many of these studies have, justifiably, focused on one or another aspect of the SARS-CoV-2 genome, such as the structural proteins that it encodes (6), its relationships to other viruses (7), and its diversity across the locations in which people have been infected (8). This leaves room for broader, more integrated approaches for the analysis of the SARS-CoV-2 genome focusing on, e.g., noncoding elements, which may yield insights missed by studies with a singular focus.

The SARS-CoV-2 pathogen is a coronavirus, and CoVs are members of the family *Coronaviridae*. *Coronaviridae* are divided into four genera based on phylogeny: alphaCoV, betaCoV, gammaCoV, and deltaCoV. CoVs have been detected in a diverse group of hosts, from humans, wild mammals (e.g., bats, pangolins, camels, civets), and birds to farm animals and poultry (9, 10). The betaCoVs are further divided into four lineages: A, B, C, and D. SARS-CoV-2 belongs to betaCoV lineage B and shares moderate genetic similarity with two human-pathogenic members, SARS-CoV (lineage B, ∼79%) and Middle East respiratory syndrome (MERS) CoV (lineage C, ∼50%), which were responsible for outbreaks of severe respiratory diseases in humans in 2002 to 2003 and 2012, respectively (11). Unlike SARS-CoV-2, SARS-CoV, or MERS CoV infection, human infection by other CoVs causes mild, common-cold-like symptoms. For example, the pathogens 229E and NL63, which belong to the alphaCoV, and pathogens OC43 and HKU1, which are within betaCoV lineage A, cause mild symptoms in humans. This suggests that genetic differences between SARS-CoV-2 and related viruses may explain its exceptional infectivity, pathogenicity, and elusiveness to effective vaccine and pharmacological mitigation strategies (12, 13).

Many noncoding elements of the SARS-CoV-2 genome have begun to receive attention as potentially informative with respect to the origins and vulnerabilities of the virus. For example, the genomic terminals of CoVs reflect noncoding 5′ and 3′ untranslated regions (5′- and 3′-UTRs) and encode conserved RNA secondary structures that have unique gene regulatory functions, as reviewed by Yang et al. (14). The UTRs are shared by both genomic and subgenomic RNAs and have been suggested to play important roles in viral replication and transcription. The UTRs can also recruit and interact with a range of host and viral protein factors and may provide long-range RNA-RNA or RNA-protein interactions through circularization of the genome. MicroRNAs (miRNAs) are evolutionarily conserved noncoding RNAs which can repress gene expression posttranscriptionally via partial sequence matches primarily to the 3′-UTRs of the target RNAs. In this light, human miRNAs can target viral RNAs and modulate different stages of the viral replication life cycle, positively or negatively (15). An example of human miRNA providing a positive influence on viral replication can be found in the hepatitis C virus (HCV), in which human-liver-specific miR-122 stabilizes the 5′-UTR of HCV, leading to the promotion of viral replication (16). Antisense oligonucleotides acting as inhibitors of miR-122 have been developed as antiviral drugs to reduce viral loads in patients (17). There are also examples of human miRNAs having the opposite effect. For example, a human miRNA showing a negative influence on viral replication (i.e., a positive effect for the host) has been reported for the influenza A virus (IAV) H1N1. Five human miRNAs that are highly expressed in respiratory epithelial cells targeting multiple gene segments have been shown to have inhibitory effects on IAV replication both *in vitro* and *in vivo* (18).

We pursued a systematic gene-by-gene comparative analysis, assessing sequence conservation in each region and element of the SARS-CoV-2 genome, including the 5′- and 3′-UTRs. We did this to see if conservation and polymorphism analyses could identify novel functional elements worth consideration in vaccine and therapeutic development. We determined whether each of these regions and elements were broadly conserved across the CoV family or unique to sublineages of CoVs. We also identified mutation hot spots, characterized the likely functional significance of naturally occurring amino acid substitutions, and assessed evidence for coevolving mutations across the genome that may impact the stability of the SARS-CoV-2 genome as a whole. Finally, we identified a unique genomic signature residing in an evolutionarily conserved element in the 3′-UTR which may be involved in host miRNA-mediated interactions and innate immunity response. These findings reveal unique viral and host conserved elements associated with the SARS-CoV-2 genome and warrant further investigation into their possible functional roles during infection as well as potential therapeutic targets.

## RESULTS

### Conserved sequence features of the coronavirus family.

**FIG 1:**
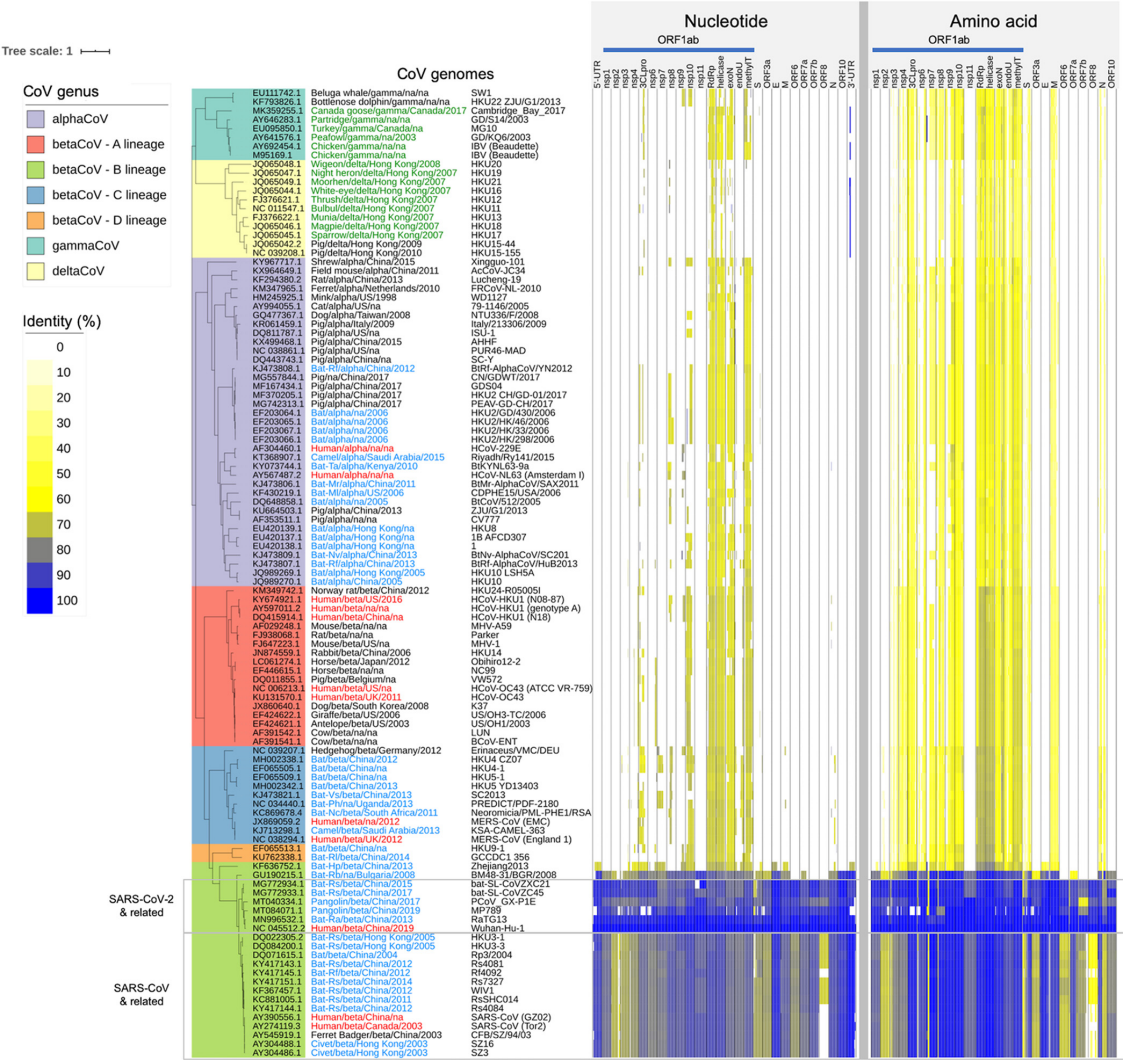
Coronavirus family genome diversity and conserved features. The coronavirus family whole-genome phylogeny, with different genera and sublineages represented, is provided on the left. Each row corresponds to a different coronavirus family member annotated with host, genus, collection location, year, and the isolate name. The CoV names are color coded to indicate host species (red, human; blue, bat, civet, camel; green, bird). The columns on the right correspond to gene products and UTR features along the length of the coronavirus genomes, with each feature normalized to the same column width. The color intensities indicate the degree of nucleotide and amino acid conservation (i.e., sequence identity) with respect to the SARS-CoV-2 reference genome (NCBI RefSeq genome accession no. NC_045512.2).

To identify conserved and potentially functional features in the CoV family, *Coronaviridae*, we compared each of the annotated genes and UTR features of the SARS-CoV-2 reference genome (NCBI RefSeq genome accession no. NC_045512.2) against 109 selected CoV family genomes (see Table S1 in the supplemental material). The SARS-CoV-2 reference isolate carries 26 processed peptides and open reading frames (ORFs), as well as two UTRs based on NCBI RefSeq annotation. The CoV family genomes that we studied were collected from four coronavirus genera (alpha, beta, gamma, and delta), including seven human CoVs (SARS-CoV-2, SARS-CoV, MERS, OC43, HKU1, 229E, and NL63), a number of mammalian CoVs (e.g., bats, pigs, pangolins, ferrets, and civets), and avian CoVs (e.g., chicken and fowls). The SARS-CoV-2 sequence features were mapped to the CoV family genome sequences through both nucleotide and amino acid sequence alignments using BLAST (19), independently of any CoV family genome annotation (Fig. 1).

10.1128/mSphere.00754-20.6 TABLE S1 CoV genomes collected from the NCBI. (A) A list of 109 CoV genomes; (B) A list of 620 betaCoV genomes. Download Table S1, XLSX file, 0.05 MB. Copyright © 2020 Chan et al. 2020 Chan et al. This content is distributed under the terms of the Creative Commons Attribution 4.0 International license.

The functional element-based conservation analysis results suggested that the 28 total genomic features (i.e., 26 processed peptides and ORFs plus two UTRs) can be broadly classified into two groups, those that were conserved across all CoV genera (cross-CoV feature group) and those that were conserved only within the betaCoV lineage B (betaCoV lineage B-specific feature group), which includes human SARS-CoV-2 and SARS-CoV, and animal CoVs from bats, pangolins, and civets. The cross-CoV feature group showed moderate levels of protein sequence identity across all genera and included nsp3-10, nsp12-16 (RNA-dependent RNA polymerase, helicase, 3′-to-5′ exonuclease, endoribonuclease, and 2′-*O*-ribose methyltransferase), and the structural proteins spike (S), membrane (M), and nucleocapsid (N) (Fig. 1). The betaCoV lineage B-specific feature group mapped uniquely to betaCoV lineage B, with no sequence similarity detected in other genera at the nucleotide or protein sequence level. The betaCoV lineage B-specific feature group included nonstructural proteins nsp2 and nsp11, accessory proteins ORF3a, ORF6, ORF7a, ORF7b, ORF8, and ORF10, the structural envelope (E) protein, and the 5′- and 3′-UTRs (Fig. 1). Among these, the five most conserved features between SARS-CoV-2 and the betaCoV lineage B isolates in descending order of average nucleotide sequence identity were the 3′-UTR, the E gene, ORF10, the 5′-UTR, and nsp10, with 97.4, 95.1, 93.8, 91.1, and 89.7% sequence identity, respectively (Table S2). A short stretch (∼30 nucleotides [nt]) of the SARS-CoV-2 3′-UTR also shared high sequence similarity with specific groups of deltaCoVs (from pigs and birds; 97%) and gammaCoVs (from chicken and fowls; 94%) (see the next section). Taken together, these results showed that the nucleotide sequence of both genomic terminals (3′-UTR and 5′-UTR) are exceptionally conserved and unique within the betaCoV lineage B isolates and therefore suggest that they are of likely functional significance for SARS-CoV-2 replication, life cycle, or sustenance.

10.1128/mSphere.00754-20.7 TABLE S2 Sequence comparison of coronavirus family genomes to SARS-CoV-2. A SARS-CoV-2 gene-by-gene BLAST analysis was performed against the CoV family genomes at the nucleotide and amino acid sequence levels. (A) Nucleotide sequence identities; (B) nucleotide sequence length coverages; (C) amino acid sequence identities; (D) amino acid sequence length coverages. Download Table S2, XLSX file, 0.1 MB. Copyright © 2020 Chan et al. 2020 Chan et al. This content is distributed under the terms of the Creative Commons Attribution 4.0 International license.

### Notable signatures in the UTRs of SARS-CoVs and related genomes.

To investigate the extent of sequence conservation within the genomic terminals of SARS-CoV-2 and related isolates, we performed a multiple-sequence alignment (MSA) analysis on 620 nearly full-length betaCoV lineage B genomes collected from the NCBI Nucleotide database, which included 361 SARS-CoV-2, 113 SARS-CoV, 75 animal CoV (e.g., bats, pangolins, civets), and 71 laboratory isolates (Table S1). The 5′-UTR (SARS-CoV-2, nt 1 to 265) was defined as the 5′ terminus, and both ORF10 and the 3′-UTR together (nt 29558 to 29903) were used for the 3′-terminal analysis. ORF10 was included in the 3′-terminal analysis because ORF10 was a predicted ORF immediately upstream of the 3′-UTR, but no ORF10 expression was detected, as reported in a comprehensive SARS-CoV-2 transcriptome analysis (20). Here, we will refer to the 3′-UTR as a 3′ genomic terminus including both ORF10 and the 3′-UTR, and all genomic coordinates will follow the SARS-CoV-2 reference isolate (NCBI RefSeq genome accession no. NC_045512.2) unless otherwise noted.

**FIG 2:**
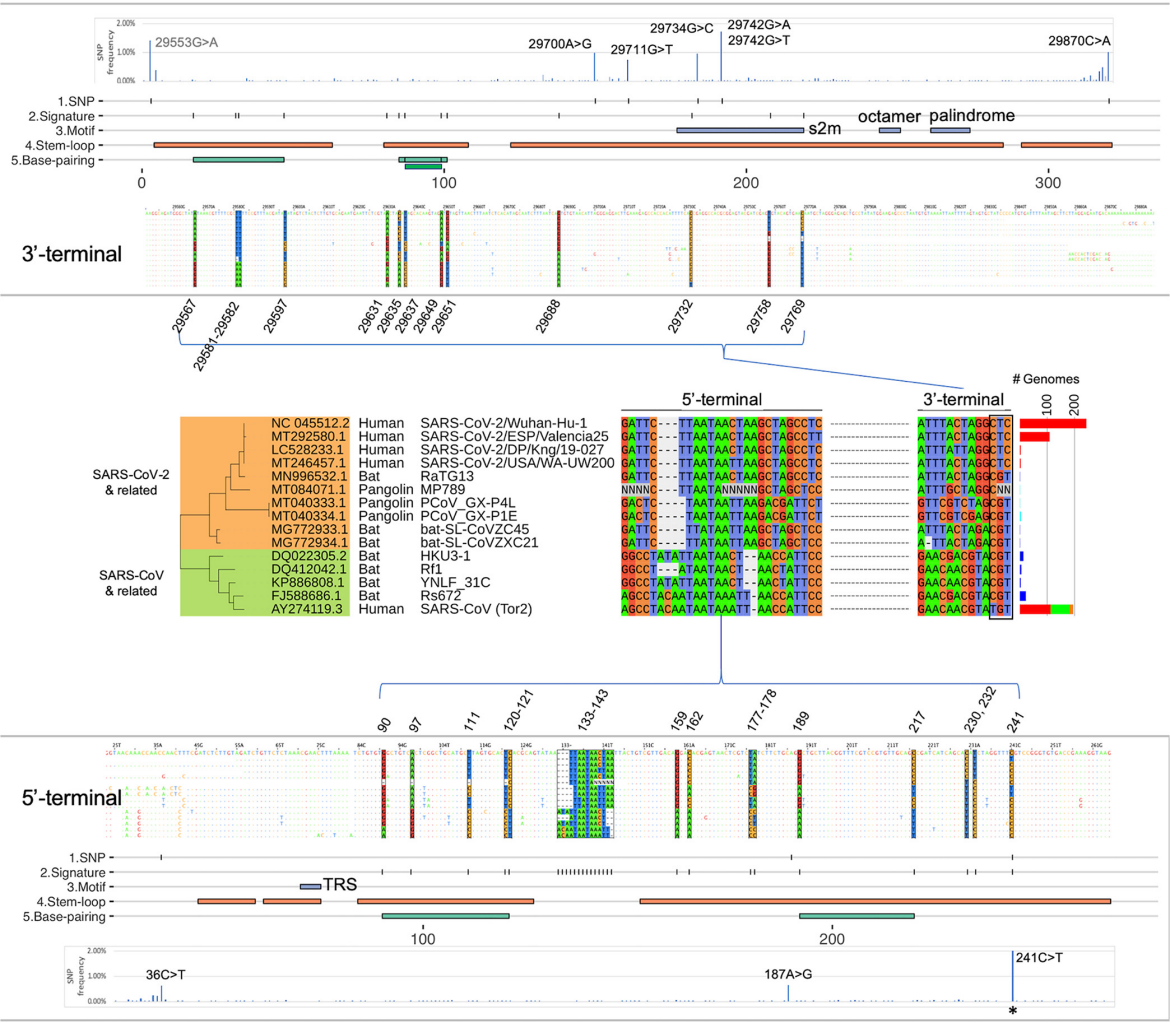
UTR signatures of betaCoV lineage B genomes. Variant positions in the SARS-CoV-2 5′- and 3′-UTRs and their presence in related SARS-CoV genomes (middle section). Base positions are color coded by the four nucleotides and depicted in their genomic locations for the 3′-UTR (top) and 5′-UTR (bottom) sequence coordinates. For each panel, the data tracks are SNV (single-nucleotide polymorphism [SNP]) frequency in SARS-CoV-2 genomes based on 18,599 GISAID genomes analyzed, SNV positions with a >0.5% mutation frequency, UTR signature positions, conserved sequence motifs, predicted stem-loops, and predicted complementary base pairings. The numbers of betaCoV genomes (# Genomes) carrying each unique signature are shown in a bar plot to the right with the following color codes for host species: red, human; blue, bat; green, laboratory; and orange, civet. The 241C>T SNV is indicated with an asterisk (*) and has an observed frequency of 70.2% (outside the frequency scale shown). The 29553G>A SNV is upstream of the 3′ terminus, with no ORF annotation showing a moderately high mutation frequency at 1.42%. s2m, coronavirus 3′ stem-loop II-like motif; TRS, transcription regulatory sequence.

The MSA analysis of the 3′- and 5′-UTRs revealed near-perfect sequence identity of the regions across the betaCoV lineage B genomes. Across the nucleotide positions where most genomes (>99%) have sequence alignments (i.e., ignoring positions near both ends of the genome, where many genomes do not have sequences), 94% of the 3′-UTR positions (234 out of 249) and 84% of the 5′-UTR positions (151 out of 179) exhibited identical nucleotides among 99% of the genomes aligned. Within these conserved regions, a high level of nucleotide diversity was observed at specific positions across the sequence alignments, with 13 and 25 hypervariable positions identified in the 3′- and 5′-UTRs, respectively (Fig. 2). These 38 positions altogether showed distinct nucleotide profiles for subclades of the betaCoV genomes, and we refer to them as the UTR “signatures.” A total of 15 major UTR signatures, as well as their frequency distribution, were identified from the 620 betaCoV genomes (Fig. 2). Based on nucleotide identities, the UTR signatures could be clustered into two distinct groups represented by the SARS-CoV-2 (Wuhan-Hu-1) and SARS-CoV (Tor2) isolates, respectively, which harbored 76% nonidentical nucleotides (29 out of 38 positions at the UTR signature positions). The UTR signature of the SARS-CoV-2 clade was shared by bat CoV isolates (RaTG13, ZC45, and ZXC21) and pangolin CoV isolates (MP789, GX-P4L, and GX-P1E), and that of the SARS-CoV clade was shared by a different group of bat CoVs (HKU3-1, Rf1, YNLF_31C, and Rs672) (Fig. 2).

Overlaying the UTR signatures with predicted RNA secondary structures revealed that a majority of the signature positions (71%; 27 out of 38) were located on stem-loop structures and that 10 positions were involved in complementary base pairings. Interestingly, we noted that the last three positions (nt 29732, 29758, 29769) of the 3′-UTR signature carried distinct nucleotide combinations for each group of the SARS-CoV-2 (CTC), SARS-CoV (TGT), and bat CoV (CGT) isolates (Fig. 2). Notably, these three positions overlapped a conserved RNA motif, S2m (coronavirus 3′ stem-loop II-like motif Rfam RF00164) previously identified in coronavirus and astrovirus (21, 22). In our analysis, the highly conserved S2m RNA element was also detectable using nucleotide searches among avian and animal CoVs belonging to the gamma and delta genera (Fig. 1). In summary, these results show that the 3′- and 5′-UTRs of SARS-CoV-2, SARS-CoV, and bat CoV isolates carry unique signatures involving predicted RNA secondary structures with likely functional and/or regulatory roles.

### UTR stability and variant sites within the SARS-CoV-2 genome.

To investigate SARS-CoV-2 genomic stability, we analyzed genome-wide nucleotide variants among isolates collected from the ongoing global outbreak. We performed single-nucleotide variant (SNV) discovery by pairwise whole-genome alignments using Nucmer on 18,599 whole-genome sequences available from the GISAID resource (as of 29 May 2020; https://www.gisaid.org) (Fig. S1, Table S3) and a set of stringent filtering criteria to identify high-confidence SNVs (see Materials and Methods). Variant analysis identified 87 variant (SNV) positions, with frequencies of >0.5% (or, equivalently, occurring in at least 93 genomes). Inspection of the UTR signature positions showed that 37 out of 38 positions were relatively stable within SARS-CoV-2 isolates, with variations detected in <0.11% genomes (i.e., 20 isolates or fewer) (Fig. 2). One exception was the variant g.241C>T, which represented one of the signature positions and was originally discovered using 361 SARS-CoV-2 genomes in the betaCoV lineage B analysis above. In this expanded analysis using 18,599 SARS-CoV-2 genomes, the variant g.241C>T was detected at a high prevalence of 70.2%. In addition, six variants were identified at five sites in the 3′-UTR (g.29700A>G, g.29711G>T, g.29734G>C, g.29742G>T, g.29742G>A, g.29870C>A) and three in the 5′-UTR (g.36C>T, g.187A>G, g.241C>T) (Fig. 2). Setting g.241C>T aside, the UTR variants were detected at a low frequency, between 0.62 and 1.05%. A very recent paper by Mishra et al. identified two variant positions corresponding to two found in this analysis in the 5′- and 3′-UTRs, respectively (i.e., g.241C>T, g.29742G>A/T) (23). In our study, all UTR variants were located on predicted stem-loop structures, with the exception of g.36C>T in the 5′-UTR. We note that position 29742 was located within the conserved RNA motif S2m and carried two alternate alleles, making it a triallelic site (Fig. 2; see Discussion). The alternate allele g.29742G>T was observed with a frequency of 1.05%, and the second alternate allele g.29742G>A was observed at a frequency of 0.67%. Based on whole-genome phylogeny analysis, the g.29742G>T and g.29742G>A variants appeared to have arisen in two distinct clades; the g.29742G>T variant was found predominantly in Asia (43% of G>T isolates), and g.29742G>A was almost equally split between Asia and North America (40.0 and 39.5%, respectively, of G>A isolates).

10.1128/mSphere.00754-20.1 FIG S1 Summary statistics based on 18,599 SARS-CoV-2 genomes from May 2020. (A) Genome distribution by region, collection month, gender, and age group. (B) Distribution of the number of SNVs detected per genome, including only variants detected in two or more genomes (i.e., excluding SNVs unique to a single genome). Download FIG S1, TIF file, 5.7 MB. Copyright © 2020 Chan et al. 2020 Chan et al. This content is distributed under the terms of the Creative Commons Attribution 4.0 International license.

10.1128/mSphere.00754-20.3 FIG S3 Linkage disequilibrium (LD) plots of coevolving variant groups using 86,450 SARS-CoV-2 genomes from October 2020. (A) LD plot of SNVs in the 9 representative coevolving variant groups identified based on 86,450 GISAID genomes, showing the squared coefficient of correlation (*r*^2^). (B) LD plots of individual coevolving variant groups. Download FIG S3, TIF file, 18.1 MB. Copyright © 2020 Chan et al. 2020 Chan et al. This content is distributed under the terms of the Creative Commons Attribution 4.0 International license.

**FIG 3:**
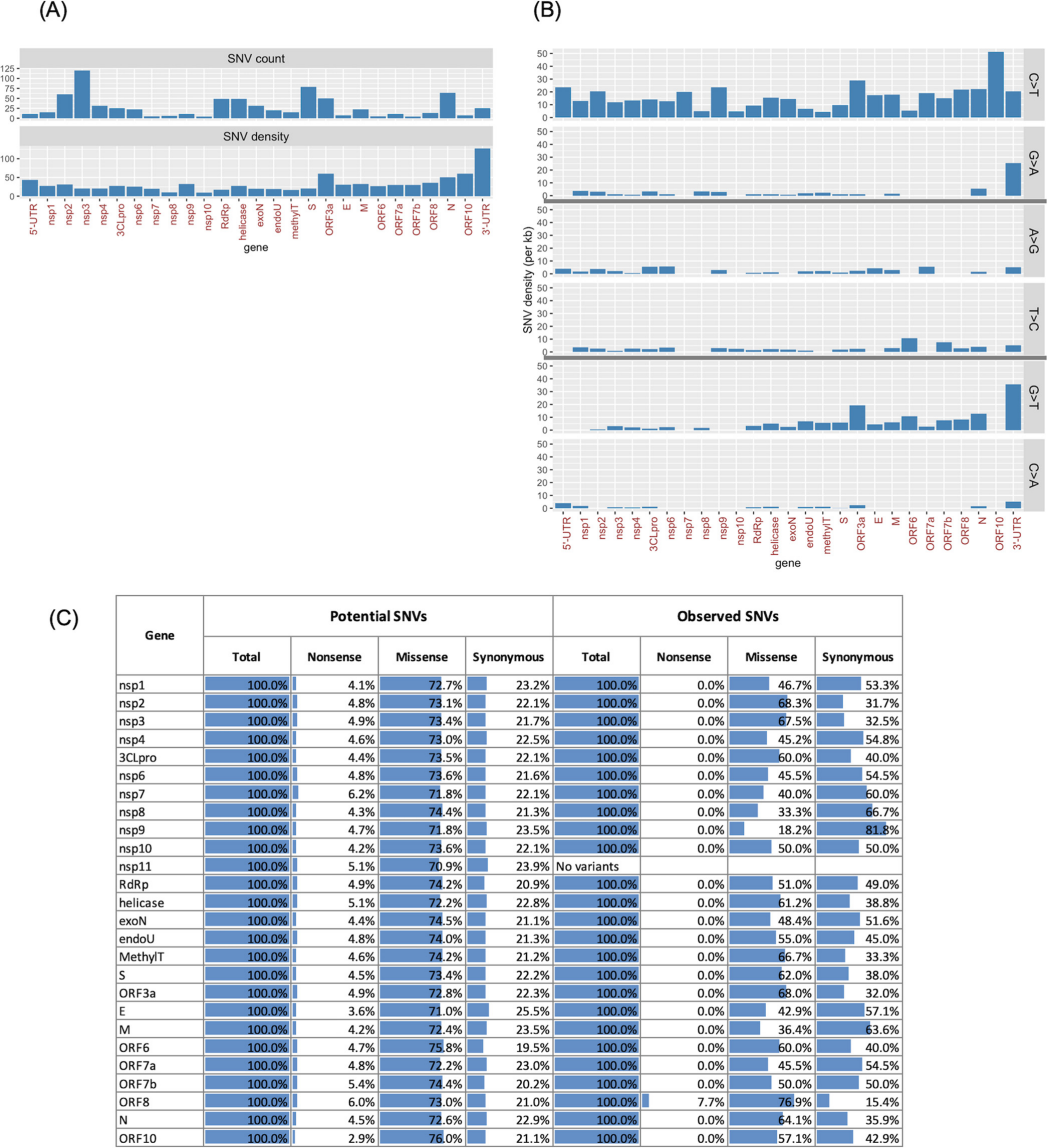
SARS-CoV-2 SNV properties. There was a total of 769 SNVs detected at a 0.05% mutation frequency of 18,599 GISAID genomes. (A) SNV counts and density (per kilobase of a feature’s length) across genes and UTRs. (B) SNV density is shown by selected base change types: C>T/G>A, A>G/T>C, and G>T/C>A. A full set of SNV distributions across all 12 base change types is shown in Table S4 in the supplemental material. (C) Amino acid mutation bias comparing expected (potential) and observed SNVs for each gene or UTR feature.

The observed SARS-CoV-2 variants were presumably the result of the evolution of the virus and potential selection pressures on those variants during the pandemic, given their likely functional impact on some aspect of the virus. Imposing a variant frequency threshold of 0.05% or higher (or, equivalently, with the variant occurring in 10 or more genomes) identified 769 SNVs (Table S4). By considering the number of variant positions per kilobase across gene features, we found that both terminal regions (3′-UTR, ORF10, N, and 5′-UTR) and ORF3a harbored the highest number of variant positions (Fig. 3A). We analyzed two aspects of the 769 SARS-CoV-2 SNVs by classifying them into types of observed base changes (i.e., A>T, A>G, A>C, etc.) and amino acid consequences (i.e., missense, synonymous, and nonsense) across the SARS-CoV-2 genes and UTRs. By assigning SNVs into different base change categories, we observed a predominance of C>T mutations out of all 12 possible base changes. The C>T mutation bias in SARS-CoV-2 has previously been suggested to be associated with human host RNA-editing activities and the subsequent fixation of the edited nucleotides in the viral RNA genome (24). The study by Di Giorgio et al. (24) pointed to C>T/G>A and A>G/T>C variants as base modification outcomes of the human APOBEC and ADAR deaminase family activities, respectively. Results from our gene-by-gene analysis confirmed the study’s observations that (i) C>T variants were the most abundant base change across almost all gene features and that (ii) C>T variants were biased toward the positive-sense RNA strand (Fig. 3B). Specifically C>T variants were more abundant than the complementary G>A variants, which would have been the complementary base change if C>T variants were to occur in the negative-sense RNA strand. Importantly, our results further revealed that the two above-mentioned properties did not hold for the 3′-UTR. In the 3′-UTR, we observed that C>T and G>A variants were more or less equally frequent and that G>T instead was the most dominant base change, followed by G>A and C>T. These results may indicate that selection pressure or regulation of the 3′-UTR was different from that of other parts of the genome. In addition, our analyses also detected G>T as the second most prominent base change type when the entire genome was considered. The gene features showing the highest density of G>T mutations were ORF3a, ORF6, the N gene, and the 3′-UTR, all of which were located in the last third of the genome. We determined that the average G>T variant density in the last third of the genome (downstream of ORF1ab) was three times higher than that in the first two-thirds of the genome (entire length of the ORF1ab) (Fig. 3B) (Fisher’s exact test, *P* = 2.6e–09). In summary, G>T variants are more enriched toward the 3′ end of the genome.

10.1128/mSphere.00754-20.9 TABLE S4 SARS-CoV-2 mutations in GISAID genomes. The list shows the mutation frequencies determined in 18,599 genomes as of May 2020 (A to D) and 86,450 genomes as of October 2020 (E to H). The descriptors include gene locations, amino acid consequences, the earliest detected isolate, and genome counts by exposure region, sex, age group, and collection month. (A, E) SNVs with a 0.05% or higher mutation frequency; (B, F) coevolving variant groups among SNVs with a 0.1% or higher mutation frequency; (C, G) indels with a 0.5% or higher mutation frequency; (D, H) SNV counts and density per genomic features for all 12 possible base change types. Download Table S4, XLSX file, 0.5 MB. Copyright © 2020 Chan et al. 2020 Chan et al. This content is distributed under the terms of the Creative Commons Attribution 4.0 International license.

To investigate whether there are any biases in terms of amino acid substitutions (i.e., missense, synonymous, and nonsense), we first determined that if an SNV occurs randomly at any given nucleotide along the genome, the chances that it results in missense, synonymous, and nonsense mutations would be 73, 22, and 5%, respectively. We also determined that such a distribution remained the same across all 26 protein-coding gene features (Fig. 3C). By analyzing the observed proportions of amino acid substitutions of the 769 SNVs, we detected fewer than expected nonsense and missense variants across all genes, with the exception of ORF8. This result likely suggested purifying selection across the protein-coding genes but not on ORF8. Furthermore, we observed that the deviations of the observed proportions from the expected values varied widely across genes (Fig. 3C). In ORF8, for example, the proportions of missense, synonymous, and nonsense variants were 76.9, 15.4, and 7.7%, respectively, which were similar to what we expected. In contrast, for the processed peptide nsp9 (whose putative function is in dimerization and RNA binding), the corresponding proportions were 18.2, 81.8, and 0%, respectively, revealing fewer missense and nonsense variants than expected. These results suggest that there is likely greatly varying selection and evolutionary pressure on individual SARS-CoV-2 genes. In the nonsense amino acid setting, only a single nonsense variant out of the 769 SNVs analyzed was detected. The variant was located in ORF8 (p.Q18*). Previous studies have identified multiple variant forms of ORF8 in SARS-CoV and SARS-CoV-related human and animal isolates (25), including a 29-nt ORF8 deletion variant that had arisen during the late-phase human transmission of SARS-CoV (26). In summary, the characterization of SARS-CoV-2 variants suggests nonrandom selection pressure, may point to undiscovered driving forces of viral genome evolution originating from the hosts or the virus, and may shed light on the identification of mutations with functional or regulatory roles.

### Analysis of SARS-CoV-2 variant combinations.

**FIG 4:**
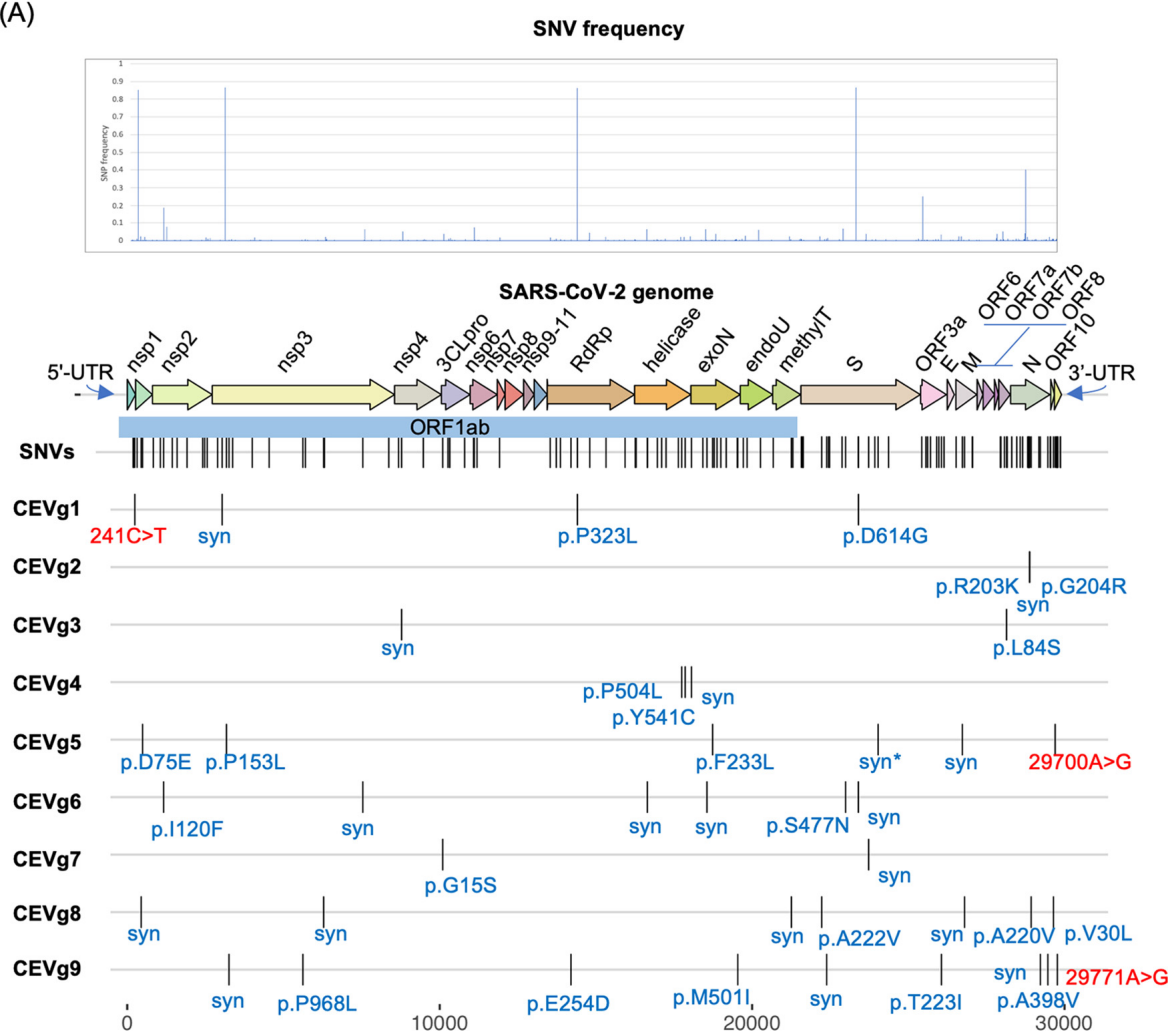

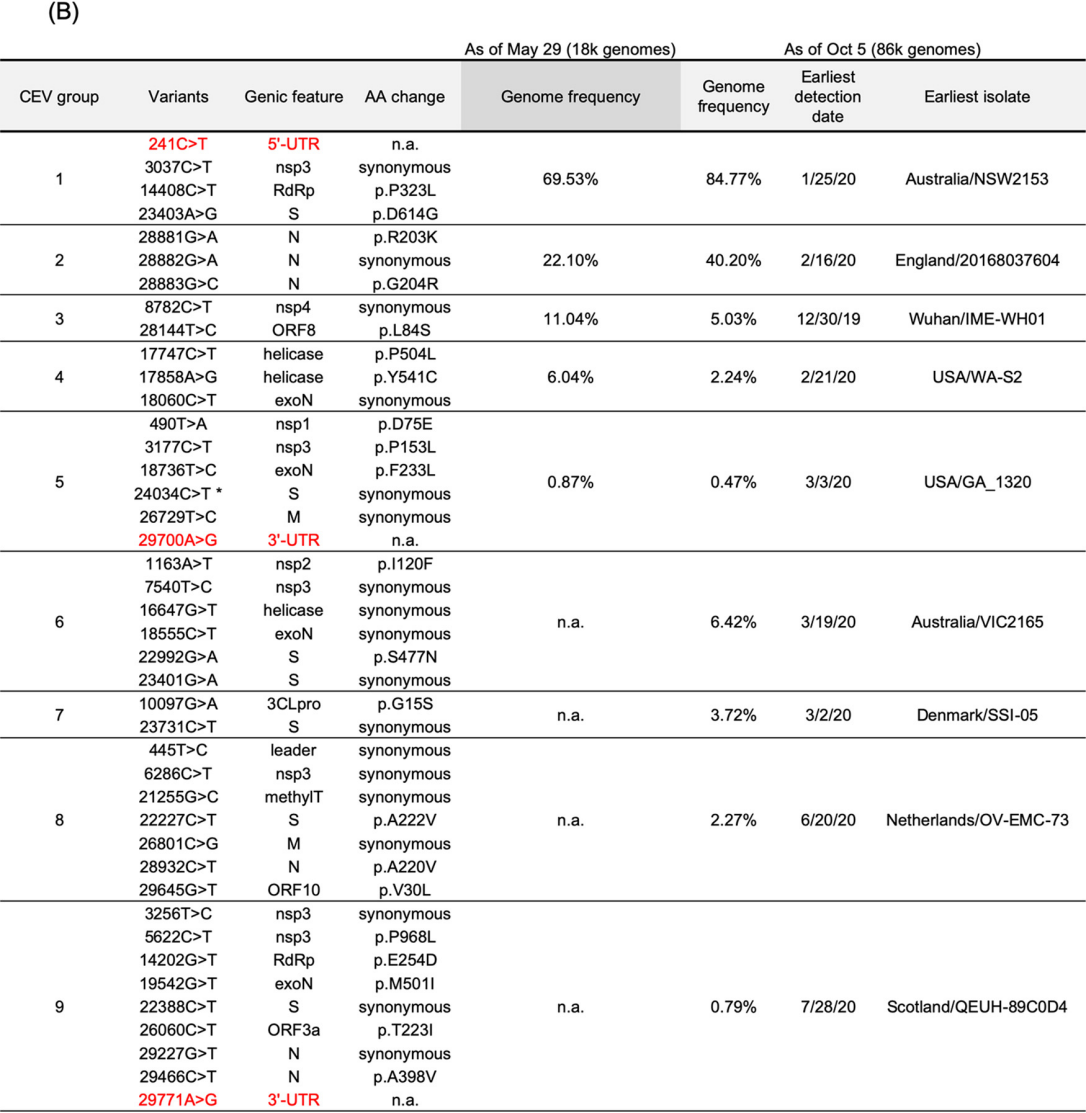
SARS-CoV-2 coevolving and independent SNVs. (A) SNV frequencies are plotted by their positions in the SARS-CoV-2 genome. The relative positions of common SNVs (>0.5%) and 9 representative coevolving variant (CEV) groups and amino acid consequences are shown. (B) Nine representative CEV groups showing different genome frequencies. Three CEV groups involved UTR variants (shown in red). syn, synonymous; RdRP, RNA-dependent RNA polymerase; exoN, 3′-to-5′ exonuclease; endoU, endoRNase; methylT, 2′-*O*-ribose methyltransferase; n.a., not applicable. *, this SNV was associated with other SNVs in CEVg5 in the 29 May 2020 data set but was no longer associated in the 5 October 2020 data set.

We performed linkage disequilibrium (LD) analysis on SNVs from 18,599 GISAID genomes collected in May 2020 using Haploview and identified a total of 34 coevolving variant (CEV) groups with a 0.1% or higher genome frequency (Table S3). Notably, we identified two CEV groups that involved the UTRs as well as other gene features, which may motivate testable hypotheses about functional dependencies or interactions of the genomic elements or regions harboring the variants. The first CEV group (CEVg1) was 5′-UTR associated, detected in 69.5% of SARS-CoV-2 genomes, and comprised of four variants that were located in the 5′-UTR (g.241C>T), nsp3 (g.3037C>T, synonymous), the RNA-dependent RNA polymerase (g.14408C>T, p.P323L), and the spike protein (g.23403A>G, p.D614G) (Fig. 4). In terms of geographic distribution by continent, CEVg1 was detected predominantly in South America (88.2%), Africa (86.8%), Europe (79.6%), and North America (66.6%), followed by Oceania (41.6%) and Asia (32.6%) (Fig. S2, Table S4). CEVg1 has shown a dramatic increase from 12.2% to 93.4% between a 3-month period from February to May 2020. The increase of CEVg1 was observed both globally and for each region by continent (Fig. S2). It has been shown that the spike protein D614G mutation, one of the variations implicated in CEVg1, is able to infect human cells more efficiently and therefore enhances transmission (6). Another CEV group (CEVg5) was 3′-UTR associated and detected in 0.9% of the genomes, and it involved six variants that resided in the leader protein or nsp1 (g.490T>A, p.D75E), nsp3 (g.3177C>T, p.P153L), the exonuclease (g.18736T>C, p.F233L), the spike protein (g.24034C>T, synonymous), the membrane protein (g.26729T>C, synonymous), and the 3′-UTR (g.29700A>G) (Fig. 4). CEVg5 was detected in a small proportion of genomes collected in North America (2.4%), Oceania (2.3%), and Europe (0.1%) but not in other regions (Fig. S2, Table S4). CEVg5 remained a minor group in March and April 2020, at 1.2 and 0.53%, respectively.

10.1128/mSphere.00754-20.2 FIG S2 Geographical and temporal distribution of genomes harboring coevolving SARS-CoV-2 variants using 18,599 SARS-CoV-2 genomes from May 2020. The proportion of genomes (*y* axis) harboring all SNVs in the CEV group among all genomes collected in each region (color) during each month (*x* axis) is shown for five coevolving variant groups and a 3-nt deletion. Download FIG S2, TIF file, 18.4 MB. Copyright © 2020 Chan et al. 2020 Chan et al. This content is distributed under the terms of the Creative Commons Attribution 4.0 International license.

10.1128/mSphere.00754-20.4 FIG S4 Geographical and temporal distribution of genomes harboring SARS-CoV-2 coevolving variants using 86,450 SARS-CoV-2 genomes from October 2020. The proportion of genomes (*y* axis) harboring all SNVs in the CEV group among all genomes collected in each region (color) during each month (*x* axis) is shown for 9 CEV groups and a 3-nt-deletion group. Download FIG S4, TIF file, 18.4 MB. Copyright © 2020 Chan et al. 2020 Chan et al. This content is distributed under the terms of the Creative Commons Attribution 4.0 International license.

10.1128/mSphere.00754-20.8 TABLE S3 SARS-CoV-2 genomes and metadata obtained from GISAID. The lists included 18,599 genomes from May 2020 (A) and 86,450 genomes from October 2020 (B), analyzed in this study. Download Table S3, XLSX file, 13.8 MB. Copyright © 2020 Chan et al. 2020 Chan et al. This content is distributed under the terms of the Creative Commons Attribution 4.0 International license.

Three additional CEV groups found in more than 5% of the genomes were identified across gene features among those genomes available as of May 2020 (Fig. 4). The first of these three, CEVg2, was detected entirely within the N protein in 22.1% of the genomes. CEVg2 consisted of three consecutive variants, g.28881G>A, g.28882G>A, and g.28883G>C, which together led to two amino acid substitutions, p.R203K and p.G204R, and the change from one to two positively charged residues. We predicted the functional impact of the two amino acid substitutions (p.R203_G204delinsKR) using PROVEAN, a prediction tool that we previously developed to determine the likely deleterious impact of amino acid substitutions and indels (i.e., nonsynonymous [Ns] coding variants) on the function of an encoded protein (27). The PROVEAN score of −2.856 suggested a deleterious effect on the protein function as a result of the two amino acid substitutions. These residues were located within a previously identified region (28) referred to as the nucleocapsid linker region (LKR; residues 182 to 247 of SARS-CoV). The LKR was identified as a flexible region joining the N- and C-terminal modular regions and included one of three intrinsically disordered regions found in the N protein; it may be involved in phosphorylation, oligomerization, and N-to-M protein interaction (28). Among the 18,599 SARS-CoV-2 genomes, the N protein also harbored the highest number of SNV counts per gene feature (i.e., 12, including coevolving and single SNVs), of which 8 were found to reside within the LKR. CEVg2 was detected in approximately one-third of the genomes collected in Europe (34.7%) and in South America (28.9%) and was also found in from 3.7 to 14.0% of the genomes in other regions. The prevalence of CEVg2 has increased in Europe (February to May 2020; 31.9 to 58.9%) and South America (February to April 2020; 0 to 36.5%) but has decreased in Asia and Africa (Fig. S2, Table S4).

The second additional CEV group, CEVg3, included two variants located in nsp4 (g.8782C>T, synonymous) and ORF8 (g.28144T>C, p.L84S) and was found in 11.0% of the genomes (Fig. 4). It has previously been reported by other groups (29, 30). CEVg3 showed geographic and temporal profiles different than those described above. CEVg3 appeared predominantly in North America (23.7%), Oceania (18.7%), Asia (17.0%), and other regions and showed a declining trend from 32.3 to 13.4 to 1.3% in January, March, and May, respectively (Fig. S2, Table S4).

The third additional CEV group, CEVg4, consisted of three variants, two in the helicase (g.17747C>T, p.P504L; g.17858A>G, p.Y541C) and one in the exonuclease (g.18060C>T, synonymous), and was detected in 6.0% of genomes (Fig. 4). Both amino acid substitutions in the helicase were predicted to be highly deleterious using PROVEAN (p.P504L score, −8.2; p.Y541C score, −8.9). Most of the genomes harboring CEVg4 SNVs (92%, 1,036 out of 1,124) were detected in North America. The per-month occurrence of CEVg4 decreased from 8.6% in February to 3.3% in April 2020 (Fig. S2, Table S4).

In addition, the processed nsp2 peptide with an unknown function carried the highest number of SNV counts (i.e., 10) after that of nucleocapsid. A moderately prevalent nsp2 mutation was detected in 22.9% of genomes (g.1059C>T, p.T85I), with a predicted deleterious functional outcome (PROVEAN score of −4.09) (Table S4). We also noted that a deletion of three consecutive nucleotides (g.1605_1607delATG), resulting in an amino acid deletion in nsp2 (p.D268del), was predicted to be deleterious (PROVEAN score of −6.370) (Table S4). This deletion of 3 nt, although identified only in a small group of 453 genomes (2.4% global collection), appeared to be highly localized in Europe (95%; 428 out of 453 positive genomes), with only a few occurrences detected in North America (7 genomes) and Oceania (14 genomes). A total of 383 genomes were collected from the following proximal regions: England (124), Netherlands (115), Scotland (102), Northern Ireland (31), and Wales (11). The prevalence of the deletion variant peaked around March in Europe (5.6%) and tapered off in April (2.2%) and May (0.7%) (Fig. S2). In all, our survey of variant positions across 18,599 SARS-CoV-2 genomes collected in May 2020 suggests that coevolving and single variants with likely functional impact on viral fitness or pathogenicity were identified across both the UTRs and functional elements throughout the genome.

In October 2020, over 86,450 high-quality GISAID SARS-CoV-2 genomes became available after our initial analyses were pursued. We have therefore updated our coevolving variant group analysis for the 86,450 genomes during the time that our research was reviewed, which is over four times the size of the first data set of 18,599, analyzed in May 2020 (Table S4, Fig. S3 and S4). A comparison of the frequencies of the CEV groups between the May and October 2020 data sets provided new insights into the SARS-CoV-2 comutation sites. First, we confirmed the global dominance of CEVg1, which carries the D614G mutation in the spike protein, and observed an increase from 69.53% to 84.77% between May and October 2020. Second, we noted the gradual disappearance (a decrease in genome frequencies) of CEVg3 and CEVg4 around July. Third, we identified two new groups of emerging coevolving mutations (CEVg6 and CEVg8) among other new groups. These two groups showed rapid increases in frequency specifically on only one continent within a short period of time and did not appear on other continents. CEVg6 emerged and increased in Oceania and increased in frequency from 0% in April to 96% in July 2020, whereas CEVg8 in Europe increased in frequency from 0% in June to 36% in September 2020. Interestingly, CEVg6 and CEVg8 each carries a new mutation in the spike protein, S477N and A222V, respectively. The A222V mutation was previously reported in a SARS-CoV-2 strain associated with a confirmed reinfection episode (31).

### SARS-CoV-2 UTRs and human miRNAs as potential therapeutic targets.

**FIG 5:**
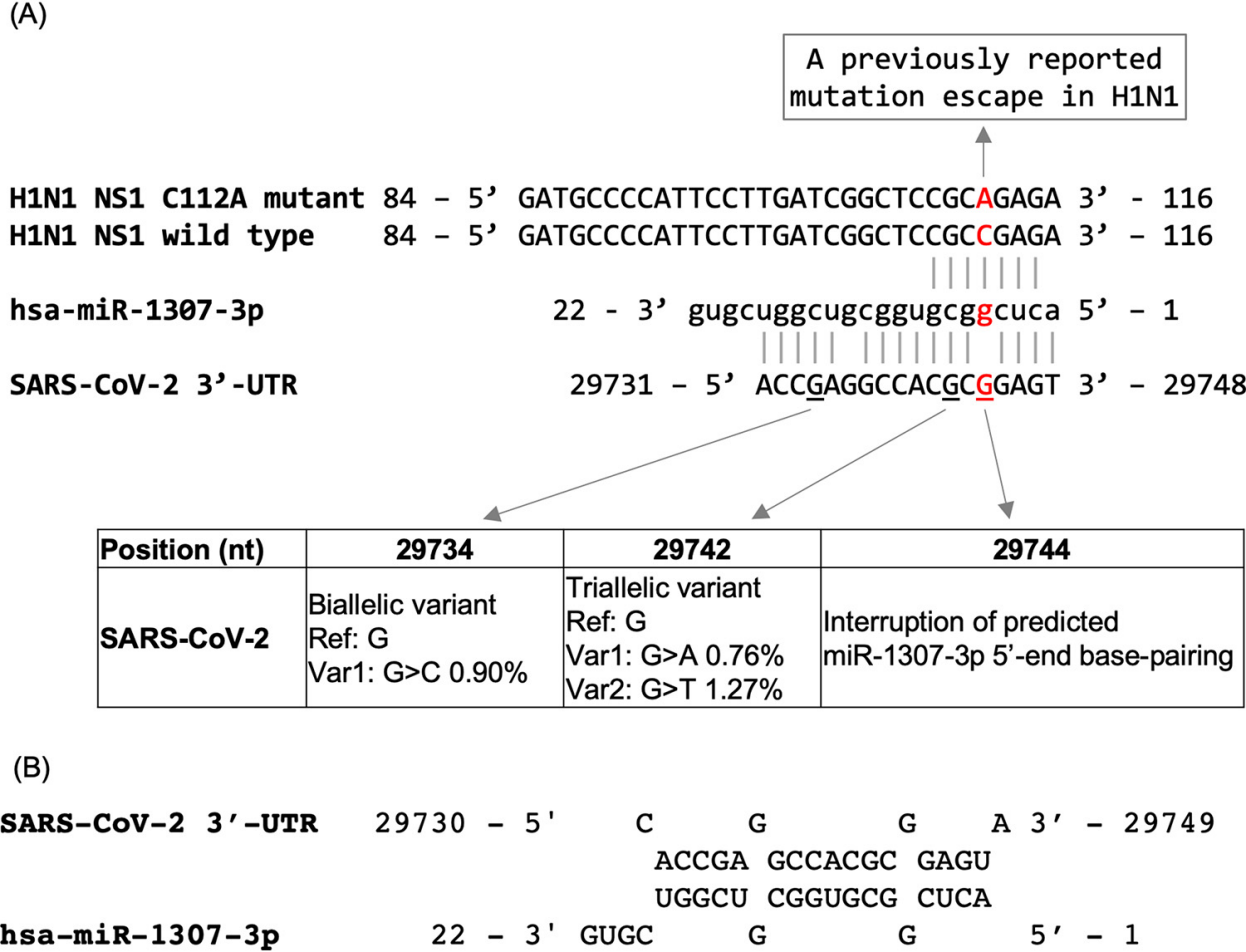
Putative human microRNA miR-1307 interaction with SARS-CoV-2. (A) Predicted base pairings between hsa-miR-1307-3p and the SARS-CoV-2 3′-UTR using blastn and search parameters for miRNAs. The base pairings of miR-1307-3p against the H1N1 NS1 C112A mutant and the H1N1 NS1 wild-type sequences were based on the work of Bavagnoli et al. (40). (B) Predicted miRNA-to-viral RNA interactions based on free energy estimates. RNA22, RNAhybrid, and IntaRNA generated consistent predictions for the RNA-RNA interaction. The prediction output from RNAhybrid is shown.

Viral UTRs and human microRNAs have been explored as therapeutic targets in HCV and other viruses because of their essential roles in viral replication and many additional functional phenomena (13). To gain insight into the possible interplay of the SARS-CoV UTRs with host microRNAs in modulating infection pathogenesis, we searched for human miRNAs sharing sequence identity with the UTR sequences of SARS-CoV-2 and SARS-CoV. We used miRNA-specific criteria for BLAST analysis for this purpose (see Materials and Methods) and identified a total of 8 and 7 human microRNAs from the miRBase database (32), including sense and antisense sequences matching the 3′- and 5′-UTRs, respectively (Table S5A). All except one miRNA-matching region (14 out of 15 miRNA regions) were located on predicted stem-loop structures (Fig. S5). Sequence matches to the human miRNAs hsa-miR-1307-3p and hsa-miR-1304-3p were located within the broader conserved RNA motif S2m. In addition to providing BLAST results tuned for miRNA searches, we provide miRNA target prediction results reported from five additional tools, including TargetScan (33), psRNATarget (34), IntaRNA (35), RNA22 (36), and RNAhybrid (37) (Table S5B). These different prediction tools exploit a combination of techniques, from nucleotide sequence-based seed matching and complement matching to structural feature characterization and free energy estimation. For miR-1307-3p, the predicted minimum energy values for RNA-RNA interactions obtained from RNA22, RNAhybrid, and IntaRNA were −31.1, −37.6, and −20.7 kcal/mol, respectively, all below the commonly considered acceptance threshold of −20 kcal/mol (Fig. 5). psRNATarget returned an expectation value (i.e., a penalty for mismatches) of 4, which was below the default and recommended value of 5. TargetScan returned no predictions for miR-1307-3p when considered against the 3′-UTR of SARS-CoV-2, as there is one base mismatch in the middle of the seed region. However, we confirmed that there is a potential interaction between miR-1307-3p and the 3′-UTR by evaluating the target prediction for a 3′-UTR variant (29744G>C). When this base change of interest was introduced at the mismatched position in the wild-type version of the 3′-UTR, a predicted miRNA target of type 7mer-m8 was reported by TargetScan. Furthermore, two recent publications reported results of *in silico* whole-genome scanning of SARS-CoV-2 to identify candidate human miRNA targets (38, 39). Khan et al. (38) applied a combination of three miRNA target prediction tools (IntaRNA, miRanda, psRNATarget) and identified a set of putative miRNAs, including miR-1307-3p for the 3′-UTR. The Khan et al. study provided additional support for a predicted target of human miR-1307-3p in the 3′-UTR of the SARS-CoV-2 genome. Importantly, a previous study of IAV H1N1 provided supporting functional evidence of hsa-miR-1307-3p in mediating antiviral responses and inhibiting viral replication (40). We discuss a possible similar role of human miR-1307-3p in SARS-CoV-2 infection below (see Discussion).

10.1128/mSphere.00754-20.5 FIG S5 Putative human microRNA interactions with the SARS-CoV-2 UTRs. (A) Predicted secondary structures of the 3′-UTR. (B) Predicted secondary structures of the 5′-UTR. (A, B) Putative human microRNA binding sites with their orientations are shown (A, antisense; S, sense). Nucleotides corresponding to the UTR signatures are in red. Sequence features of unknown functions (octamer and palindrome) in the 3′-UTR and the TRS conserved element in the 5′-UTR are shown in blue. The S2m motif is indicated by two inverted triangles. Download FIG S5, TIF file, 10.4 MB. Copyright © 2020 Chan et al. 2020 Chan et al. This content is distributed under the terms of the Creative Commons Attribution 4.0 International license.

10.1128/mSphere.00754-20.10 TABLE S5 Putative human miRNAs showing sequence similarities with SARS-CoV or SARS-CoV-2 UTRs. (A) List of human miRNAs sharing sequence identities with the UTRs of SARS-CoV and SARS-CoV-2. (B) miRNA target predictions using multiple tools. n.p. refers to no predictions obtained. (C) Human miRNA tissue expression levels collected from the IMOTA database. (D) miRNA expression in Calu-3 human lung cells. Data were sourced from GEO accession no. GSE148729 (44). The fold change for SARS-CoV-2 infection samples was calculated in comparison with the mock infection samples based on count per million values. Ranks show the ranks of the miRNAs among all miRNAs in terms of average expression level in counts per million. (E) Human miRNA cross-species conservation analysis. The table shows the number of miRNAs detected in other organisms in the miRBase database grouped by taxonomic class. Note that search conditions required 18 or more matching nucleotides and allowed gaps, and the number includes the human miRNA itself. Download Table S5, XLSX file, 0.02 MB. Copyright © 2020 Chan et al. 2020 Chan et al. This content is distributed under the terms of the Creative Commons Attribution 4.0 International license.

We also examined the endogenous expression of the 15 identified miRNAs using the human miRNA tissue atlas IMOTA (41), which provided categorized miRNA expression levels (i.e., high, medium, low, or not expressed) for 23 human tissues (Table S5C). Among the 8 miRNAs with expression data available, three miRNAs (hsa-miR-1307-3p, hsa-miR-1304-3p, and hsa-miR-15b-5p) were reported to be expressed mostly at medium level in all 23 tissues, including lung, heart, liver, kidney, and small intestine, some of which tissues have been reported to be severely affected during the SARS-CoV-2 infection (42, 43). The expression of miR-1307-3p upon SARS-CoV-2 infection was obtained from the Wyler et al. study (44) using the human lung cell line Calu-3 (GEO accession no. GSE148729). From the raw read count data, we determined the trimmed mean of M (TMM) value-normalized expression levels (45) of miR-1307-3p for mock infection and postinfection to be 362.2 and 485.3 cpm, respectively (Table S5D). The expression level of miR-1307-3p increased slightly by 1.3-fold across 4 to 24 h postinfection compared to that after mock infection. Furthermore, we searched the miRBase database to determine whether the 15 identified human miRNAs were conserved in other organisms. While 6 miRNAs were not detected in other organisms, 9 miRNAs were found in a number of other mammalian species, with the number of organisms ranging from 3 to 25 (Table S5E). The hsa-miR-1307-3p miRNAs, for example, have been found in 12 other mammalian species in various taxonomic orders, such as primates (e.g., orangutan, chimpanzee, baboon, aye-aye), Artiodactyla (e.g., pig, goat, cow), and others (e.g., bat, dog, rabbit, horse, armadillo). SARS-CoV-2 viral sequences have been detected in dogs from households with confirmed human cases, but the dogs remained asymptomatic (46).

In summary, these results suggest that the noncoding UTRs of SARS-CoV-2 are made up of sequences that, based on base pairing, complementarity, and interaction analyses, may interact with microRNAs in humans or other species. Further functional assays are needed to delineate whether and how microRNAs are involved in the modulation of viral replication and pathogenesis.

## DISCUSSION

Our SARS-CoV-2 genome-wide analyses demonstrate that ultraconserved 5′- and 3′-terminal regions of SARS-CoV-2 are shared among betaCoV lineage B genomes, including SARS-CoV and different groups of bat CoVs; however, genome-wide genetic similarity may be as low as ∼79%. Notable UTR variant signatures, including complementary base pairing positions with encoded secondary structures, were identified from representative genomes. The high degree of primary sequence conservation of the UTRs identified in this study and the predicted RNA secondary structures reported in two recent studies (47, 48) provide strong evidence for conserved functions of the UTRs in the betaCoV lineage B SARS family of viruses. The likely participation of UTRs in long-distance RNA-RNA and/or RNA-protein interactions involving viral and host factors in the replication of CoVs has been proposed and is consistent with our study results; it therefore deserves greater attention (14).

In addition, our gene-by-gene comparative analysis of the CoV family provided an account of sequence conservation and dissimilarities in both nucleotide and amino acid aspects across each functional unit (processed peptides, ORFs, and UTRs) of the SARS-CoV-2 genome. The CoV family reference genomes were collected from multiple sources, including NCBI RefSeq (49) and previous CoV studies (50, 51), and therefore represent a broad collection of all CoV genera (alpha, beta, gamma, and delta), host species (humans, mammals, and birds), and disease outcomes (human or farm animal outbreaks or mild symptoms). We believe that our genome-wide sequence analysis is complementary to conventional MSA and phylogenetic analyses (e.g., gene tree) (4) or localized window-based analyses (e.g., Simplot) (2), which have been used to assess genome/gene sequence conservation. The cross-CoV conservation data generated in this study will provide the basis for a range of follow-up studies, such as determining the functional significance of highly conserved genes and domains (e.g., the E protein), designing vaccine candidates based on protein or RNA conservation, and developing lineage-specific diagnostic markers for community monitoring and interspecies tracing.

Our analyses also suggest that naturally occurring variants in the SARS-CoV-2 genome sequence were relatively low, with approximately 0.3% of sites exhibiting variations if one imposes a 0.5% or higher mutation frequency threshold. This is consistent with a low mutation rate of the SARS-CoV-2 RNA-dependent RNA polymerase, which likely possesses a proofreading function similar to that of SARS-CoV (52). The observation that the SARS-CoV-2 UTRs harbored higher frequencies of natural variations (3′-UTR, 2.6%; 5′-UTR, 1.2%) than the overall genome-wide mutation rate of 0.3% was likely due to lower evolutionary constraints present in the noncoding UTRs than in genes in the protein-coding regions. A recent report suggesting the influence of human RNA-editing activities on viral genome mutations has provided some explanations for the overall mutation biases that we observed (i.e., the C>T substitution predominance) (24).

Identifying possible therapeutic targets in noncoding regions of a genome has been pursued with other RNA viruses (13), and our investigations suggest possible SARS-CoV-2 UTR interactions with human miRNAs. We used a bioinformatics approach to identify genomic regions sharing strong sequence identity (≥18 nt) to human miRNAs as represented in miRBase (32). Because the mature miRNAs can recognize and bind to a target RNA site through canonical or noncanonical matching positions, our initial analyses used sequence identity as an all-inclusive guiding parameter for the human miRNA screen. We have also attempted to generate predictions from five additional orthogonal miRNA target prediction tools utilizing seed matching, complement matching, structural features, or free energy estimation and included additional supporting evidence for predicted miRNA-virus interactions.

We identified a putative hsa-miR-1307-3p binding site in the 3′-UTR of SARS-CoV-2 with strong sequence identity that exhibits 16 nt of Watson-Crick base pairings out of the first 18 nt of the miRNA (Fig. 5). The putative binding site spanned a conserved RNA motif, S2m, which was also found in the 3′-UTR of subsets of betaCoVs (e.g., SARS-CoV), gammaCoVs (e.g., infectious bronchitis virus from chicken), and deltaCoVs (e.g., birds, pigs). The S2m motif had been previously identified as a conserved element in other CoVs and astrovirus (21, 22). For some of the CoV genomes, due to a lack of high-quality sequences available from the genomic terminals (i.e., nonambiguous bases), the actual frequency or taxonomic distribution of the S2m and other conserved RNA elements present in the UTRs may have been underestimated. Ongoing efforts to collect and whole-genome sequence the repertoire of naturally occurring CoV isolates from wild animals, including bats (53), should help to shed new light on the evolution of CoV functional elements.

Previous studies have associated hsa-miR-1307-3p miRNA with cancer progression as well as lung function. miR-1307 was originally discovered as a novel human miRNA upregulated in Epstein-Barr virus (EBV)-positive nasopharyngeal carcinomas (54) and was also suggested to be associated with the progression of prostate cancer (55). miR-1307 expression has been shown to be dysregulated in newborns with chronic lung disease (56). Importantly, the study by Bavagnoli et al. demonstrated a functional role of miR-1307 in the regulation of viral replication in the influenza A virus H1N1, which was the pathogen responsible for the 2009 H1N1 pandemic (40). Their study predicted sequence complementarity of miR-1307 to H1N1 nonstructural protein 1 (NS1), which functions to limit interferon and proinflammatory responses, thus allowing the virus to evade host innate and adaptive immunity and replicate efficiently in infected cells. The same study also showed that miR-1307 overexpression had regulatory effects on both the virus and host cells. First, miR-1307 overexpression was able to reduce NS1 expression and inhibit wild-type H1N1 replication but had no effects on the NS1 C112A mutant, which carried a nucleotide mismatch to the 5′ region of miR-1307 (Fig. 5). Second, the overexpression of miR-1307 (in a stably transfected lung cell line) was able to induce genes involved in cell proliferation, apoptosis, and the regulation of inflammatory and interferon responses. Taken together, the study concluded that the C112A variant was a viral escape mutation for miR-1307 regulation. Furthermore, the study reported that the C112A mutant was significantly associated with the severe clinical symptom acute respiratory distress syndrome and represented close to one-third of influenza strains that circulated primarily locally in northern Italy during the 2010–2011 influenza season.

In SARS-CoV-2, it is notable that an interruption of base pairings from nt 29744 to the 5th position of the miR-1307-3p sequence coincides with the location of the C112A mutation in H1N1 (Fig. 5). It can be hypothesized that SARS-CoV-2 shares a common host defense mechanism with H1N1, that this mechanism is mediated by host cellular miRNA regulation, and that SARS-CoV-2 carries an allele whose regulation is weakened by human miR-1307 because of the nucleotide mismatch. In support of this hypothesis, our population analysis of SARS-CoV-2 variations identified two nearby mutations at positions 29742 and 29734, which correspond to the 7th and 15th positions of miR-1307, respectively. Mutations that occurred at these two sites may presumably further disrupt the hypothesized base pairings with miR-1307 to escape from binding and inhibition. So far, as of October 2020, the mutations were detected at a low frequency (<1.2%) in the ongoing outbreak. In all, whether SARS-CoV-2 and H1N1 infections have similar host defense mechanisms mediated by host miRNA regulations or whether human population variations of hsa-miR-1307-3p are associated with the severity of clinical symptoms are presently not known and warrant further investigation.

In summary, we utilized a comprehensive genomic analysis approach to assess sequence variations of the SARS-CoV-2 genome with respect to the coronavirus family as well as circulating strains during the current global outbreak collected via the GISAID repository. We pursued these analyses to gain insights into functional elements within the SARS-CoV-2 viral genome. We identified distinct viral clades sharing coevolving sequence variants and explored emergence and global spread by continent and collection time. We identified possible interactions of the human microRNA miR-1307-3p with the noncoding 3′-UTR of the SARS-CoV-2 genome supported by *in silico* predictions from this study, new analyses from other groups (38, 39), and extensive functional assays that supported a biological role for miR-1307-3p in H1N1 influenza A virus replication (40). Above all, because of the challenges of canonical and noncanonical properties of miRNA binding to targets, we note that important next steps are functional experiments, such as miRNA-virus biochemical interaction assays, mutational analysis, and miRNA overexpression assays to further investigate the biological significance of miR-1307 *in vitro* and *in vivo* during SARS-CoV-2 replication and possibly the regulation of host immune responses. Through this work, we provide evidence for and insights into the possible involvement of miR-1307 in SARS-CoV-2 infection and, consequently, new opportunities for exploring potential targets for antiviral interventions.

## MATERIALS AND METHODS

### Coronavirus family sequence conservation analysis.

The SARS-CoV-2 NCBI RefSeq genome (NC_045512.2) was used as the reference. For gene-by-gene analysis, each sequence of 28 annotated genomic features (ORFs, processed peptides, and UTRs) of SARS-CoV-2 was searched against the 109 representative CoV genomes collected from four genera (alpha, beta, gamma, and delta) (Table S1) using NCBI BLAST+ (blastn and tblastx; v2.9.0), with an E value threshold of 1e–3. The MSA of the 109 CoV family genome sequences was performed using Clustal Omega (v1.2.4) (57). The maximum likelihood phylogeny tree was constructed using RAxML (v8.2.11), with 100 bootstraps under the GTRGAMMA model (58). The tree was visualized using iTOL (59).

### SARS-CoV-2 genomic terminal sequences.

In the context of this study, the 5′ terminus (nt 1 to 265) corresponded to the annotated 5′-UTR. The 3′ terminus (nt 29558 to 29903), which was also denoted 3′-UTR, corresponded to the annotated ORF10 and 3′-UTR of the SARS-CoV-2 reference genome (NCBI RefSeq genome accession no. NC_045512.2).

### Collection of betaCoV lineage B genomes and UTR analysis.

A total of 693 betaCoV genome sequences were initially collected from the NCBI Nucleotide database (as of 15 April 2020). Genome sequences were collected using the entire SARS-CoV-2 genome sequence as the query for a blastn search, which required that most of the query sequence length and both UTR regions be aligned sufficiently for sequence comparison (i.e., that at least 85% of the query sequence was covered; an alignment starting from nt 130 or a smaller nucleotide position exists, and an alignment ending at nt 29700 or a higher nucleotide position exists). An MSA was performed on the collected 693 genome sequences, including the SARS-CoV-2 reference genome, using Clustal Omega (v1.2.4). For the 3′- and 5′-UTR regions, variable positions were defined as any positions where 5% or more of the genomes showed nucleotide differences from the reference (excluding ambiguous nucleotides, such as N nucleotides). Positions near either end of the genome (i.e., nucleotides below position 87 or above position 29806) were excluded since over 1% of the genomes do not have aligned sequences and therefore the MSA may not be of high quality. Finally, after the genomes with ambiguous nucleotides in the defined variable positions in UTRs were filtered out, 620 genomes were used as the final genome set for UTR signature analysis. Note that a pangolin CoV (MT084071.1) was included in spite of its having ambiguous nucleotides because it appeared to be one of likely close relatives of SARS-CoV-2 and also carried a unique UTR signature.

### Prediction of the UTR secondary structure.

RNA secondary structure prediction was performed using the RNAfold Web server (http://rna.tbi.univie.ac.at/cgi-bin/RNAWebSuite/RNAfold.cgi) with the default basic option to calculate the minimum free energy (MFE) and partition function. The predicted SARS-CoV-2 5′- and 3′-UTR structures previously reported in reference 4 were used to adjust the prediction.

### SARS-CoV-2 variant analysis.

A total of 34,217 SARS-CoV-2 genome sequences and their associated metadata were obtained from GISAID (https://www.gisaid.org/) on 29 May 2020. A data sanitization and filtering step was performed, and it included removing gaps (dash and space characters), filtering out genomes from a nonhuman host, and keeping only high-quality genomes (i.e., requiring a genome to be longer than 29 kb and to contain <1% Ns and no other ambiguous nucleotides, such as B and W). Each of the remaining 18,599 high-quality genomes was aligned with the reference genome to identify variants using the nucmer and show-snps functions of the MUMmer package (v3.23) (60). Sequence variants identified within the poly(A) tail or near either end of the sequence (within 10 nt from either end) were ignored. In addition, an MSA of the 18,599 genomes was built using MAFFT (v6.861b), which was used for independent validations of major mutation positions (61). For each sequence variant, the mutation effects on gene products (i.e., genic location and amino acid change, if applicable) were analyzed using in-house scripts. The functional impacts of amino acid substitutions and indels were predicted using PROVEAN (27). Linkage disequilibrium (LD) analysis was performed to identify coevolving variants among SNVs with a frequency of 0.1% or higher using Tagger, implemented in Haploview (v4.2) (62), and using the squared coefficient of correlation (*r*^2^) threshold of 0.8. Non-biallelic sites needed to be excluded from the LD analysis, and a set of 140 genomes with rare mutations on the major mutable sites, causing the sites to become non-biallelic, were also excluded. During the revision of the manuscript, we repeated the same analyses using an up-to-date (as of 5 October 2020) data set with 135,500 genomes. After the same filtering steps, 86,450 genomes were included for the analyses, and the new findings in the coevolving variants group analysis were also reported.

### Protein-coding SNV analysis.

Each of the identified protein-coding SNVs was analyzed to determine its amino acid consequence (missense/synonymous/nonsense) using in-house scripts. For the estimation of amino acid consequences under the assumption of random mutations (i.e., to enumerate all potential SNVs given the sequence context of the SARS-CoV-2 genome), all 3 possible SNVs for every nucleotide position on all coding sequences from the start codon to the last codon before the stop codon were included in the analysis.

### Identification of putatively interacting human microRNAs.

The UTR sequences of SARS-CoV-2 and SARS-CoV were used to search against the miRBase mature RNA sequences (release 22.1) (32) using blastn, with the following parameters set for short sequences: “-penalty -4 -reward 5 -gapopen 25 -gapextend 10 -dust no -soft_masking false.” For cross-species conservation analysis of other organisms, we searched the miRBase database with a requirement of 18 or more bases matched with 100% sequence identity. For the additional five miRNA target prediction tools, the results were obtained using the following downloaded scripts or corresponding Web servers with the default parameters: TargetScan, http://www.targetscan.org/vert_72/vert_72_data_download/targetscan_70.zip; psRNATarget, http://plantgrn.noble.org/psRNATarget/analysis?function=3; IntaRNA, http://rna.informatik.uni-freiburg.de/IntaRNA/Input.jsp; RNA22, https://cm.jefferson.edu/rna22/Interactive/; and RNAhybrid, https://bibiserv.cebitec.uni-bielefeld.de/rnahybrid.

### Statistical analysis.

To test for the significance of the G>T mutation bias toward the 3′ end of the genome, the proportions of G>T mutations out of summed gene lengths were compared between ORF1ab (60 mutations out of 21,326 nt) and the remaining ORFs (66 mutations out of 7,974 nt) using Fisher’s exact test implemented in the fisher.test function in the R stats package (v3.6.1).

### Data availability.

Genome sequence data are available through the NCBI and GISAID.
