# Supplementary figures and images for: Conserved Genomic Terminals of SARS-CoV-2 as Coevolving Functional Elements and Potential Therapeutic Targets
Source: mSphere. 2020 Nov 25;5(6):e00754-20. doi: 10.1128/mSphere.00754-20 (PMC7690956; doi:10.1128/mSphere.00754-20)

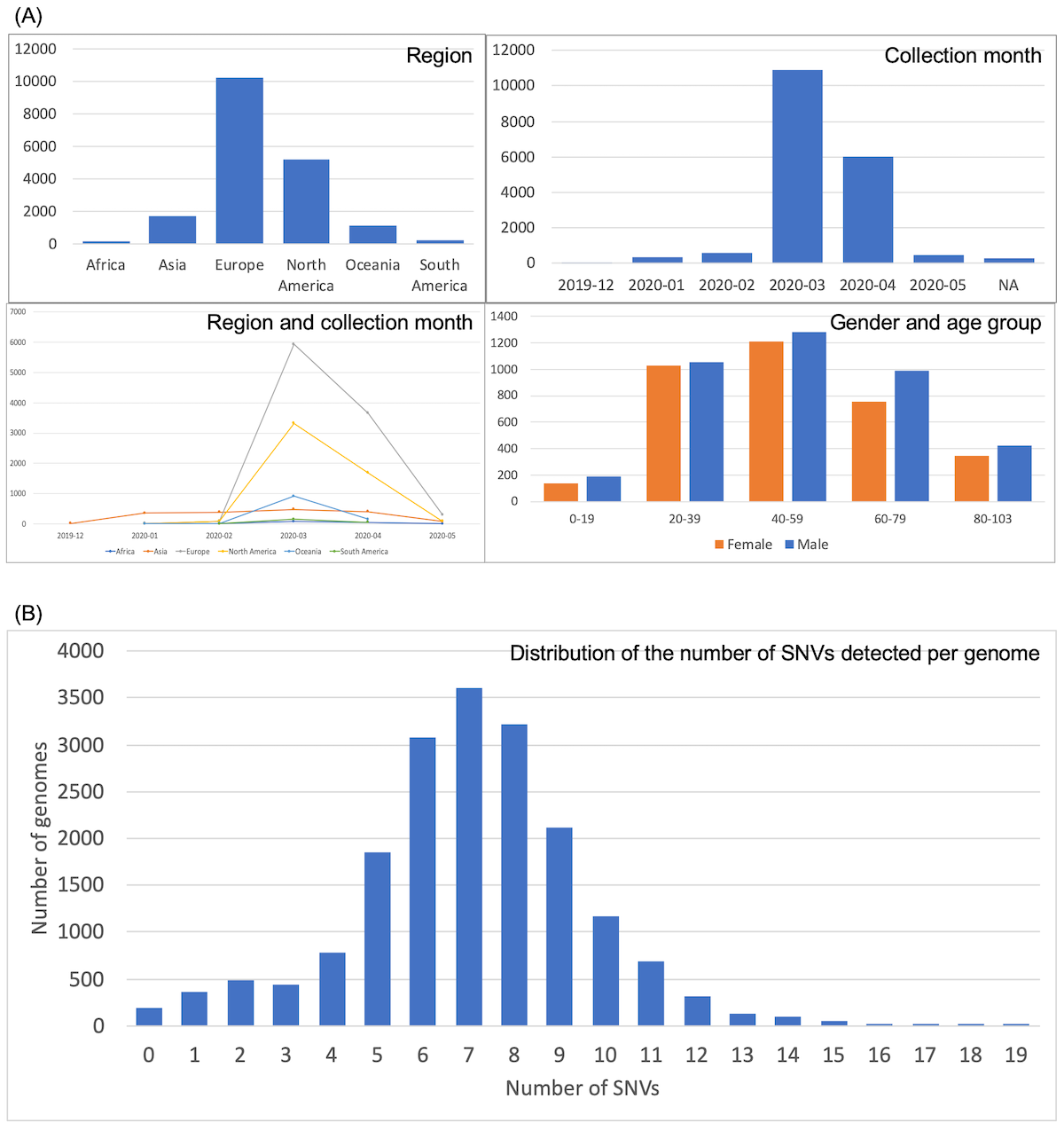

Supplement: FIG S1 [file mSphere.00754-20-sf001.tif]

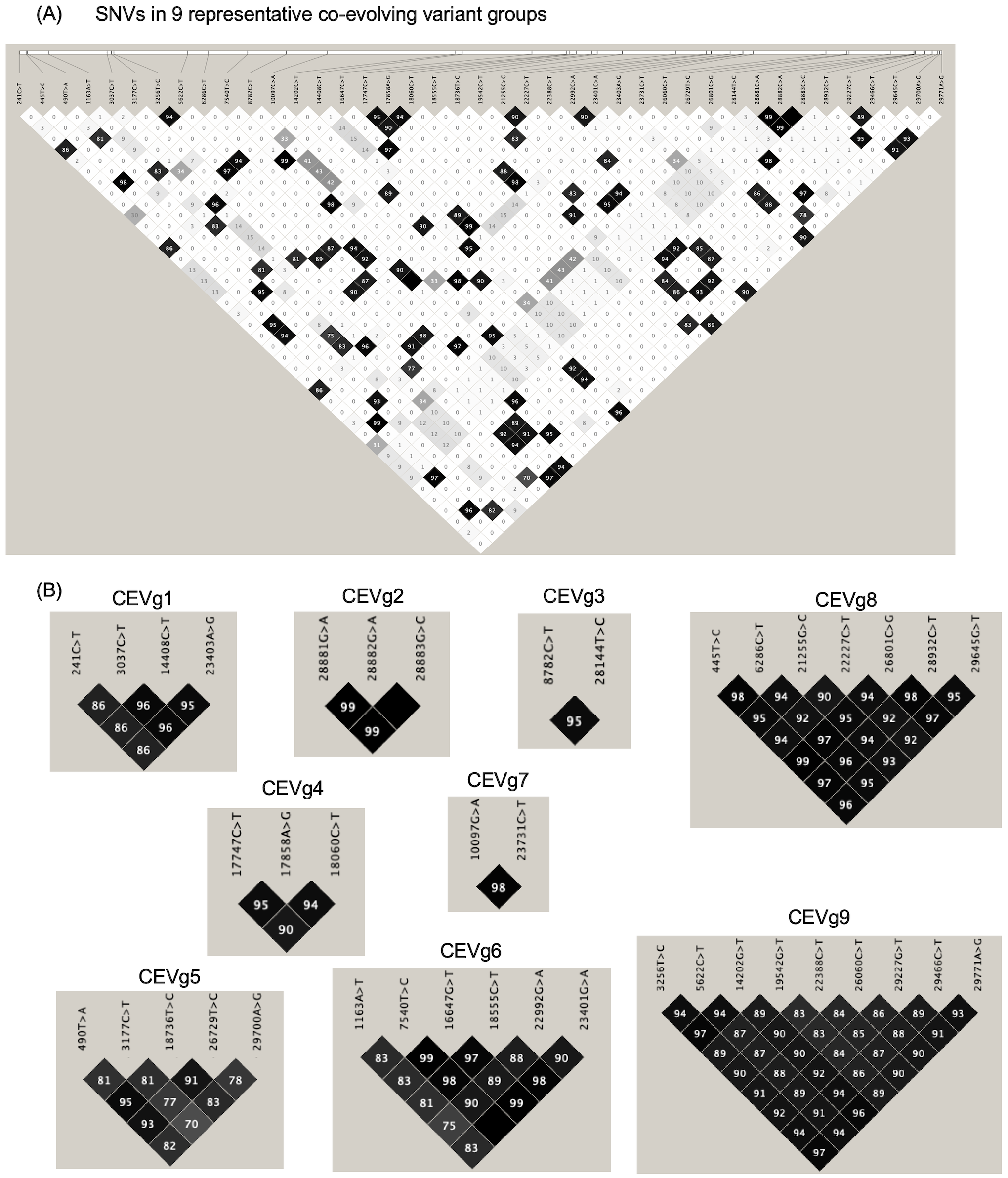

Supplement: FIG S3 [file mSphere.00754-20-sf003.tif]

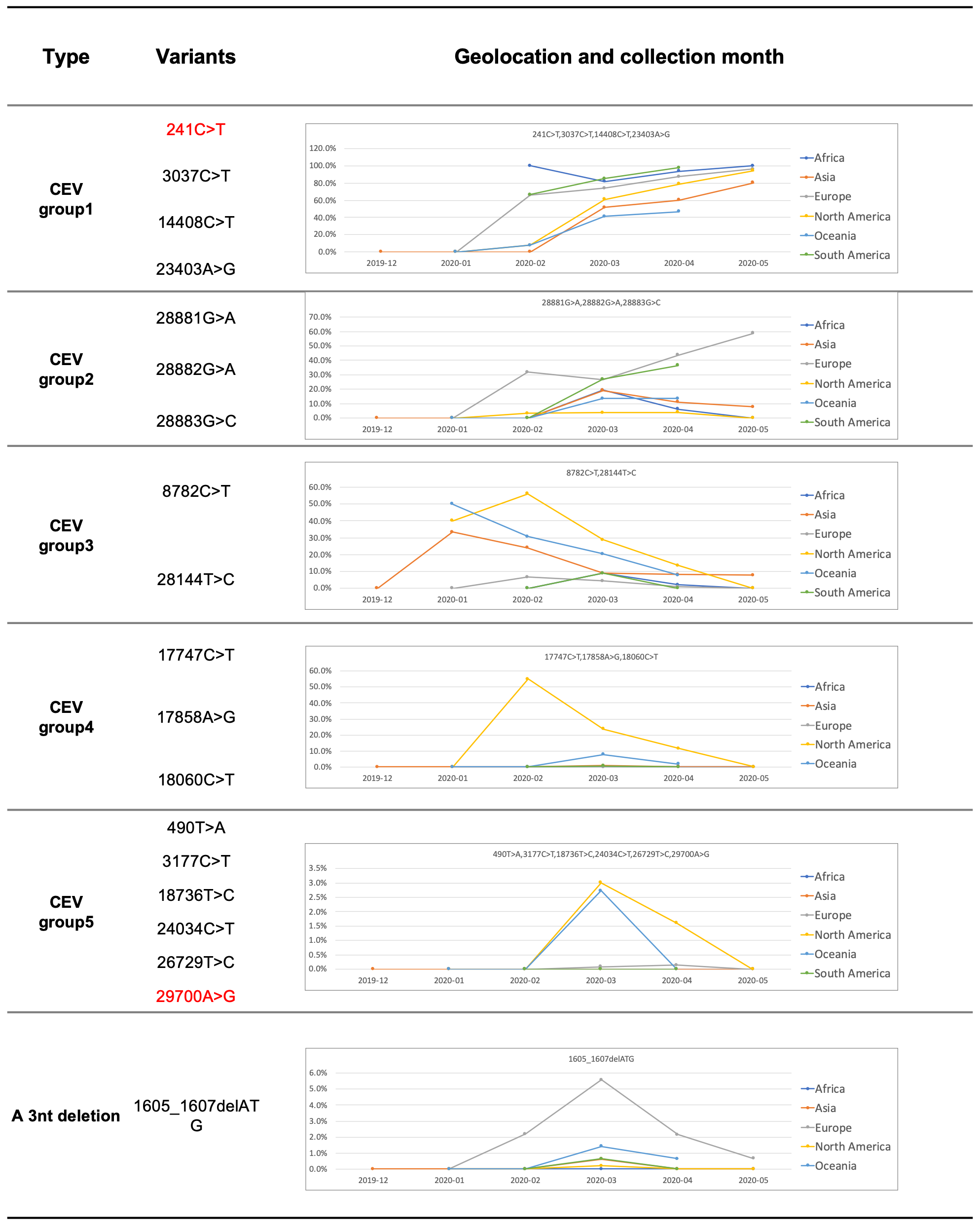

Supplement: FIG S2 [file mSphere.00754-20-sf002.tif]

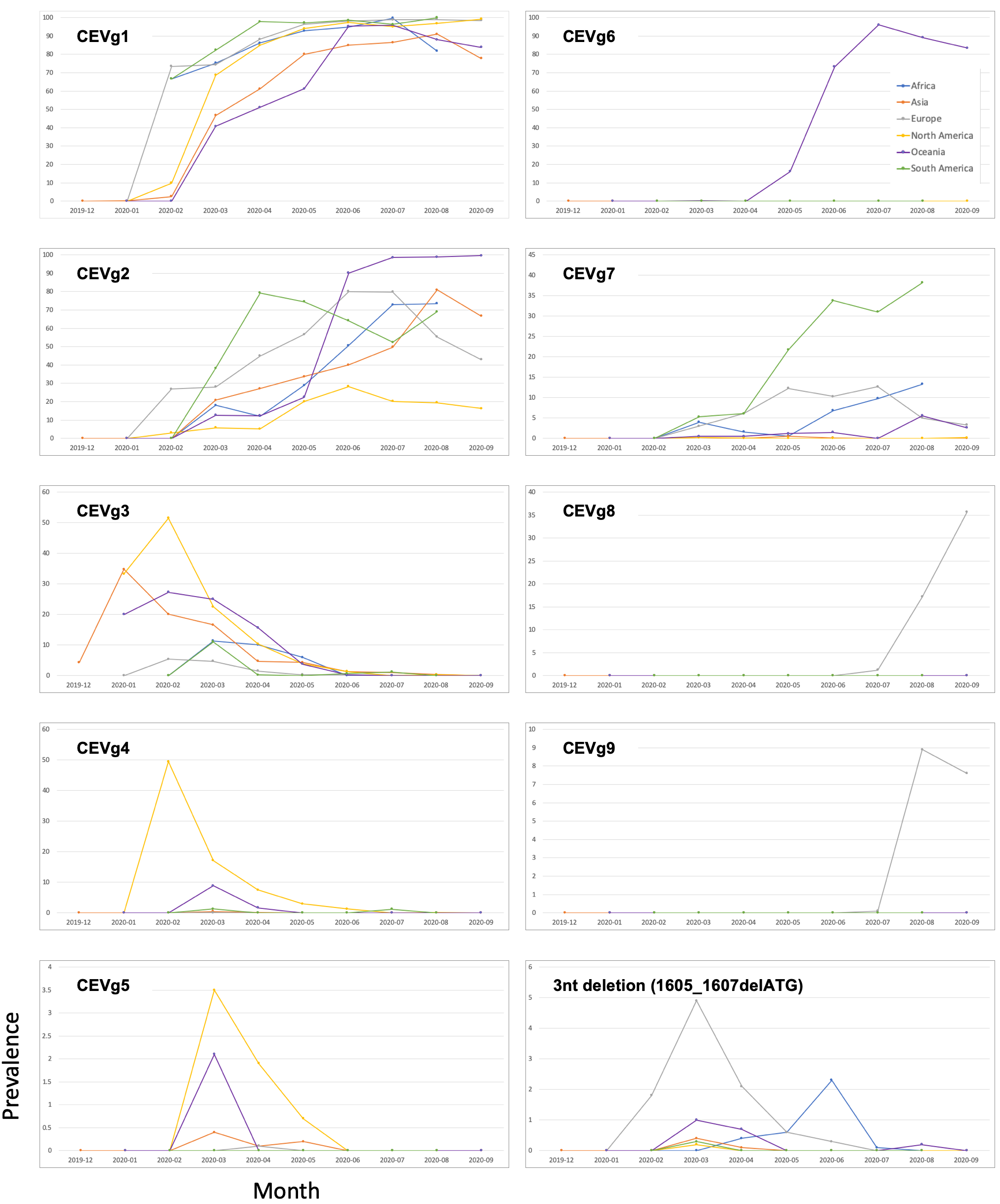

Supplement: FIG S4 [file mSphere.00754-20-sf004.tif]

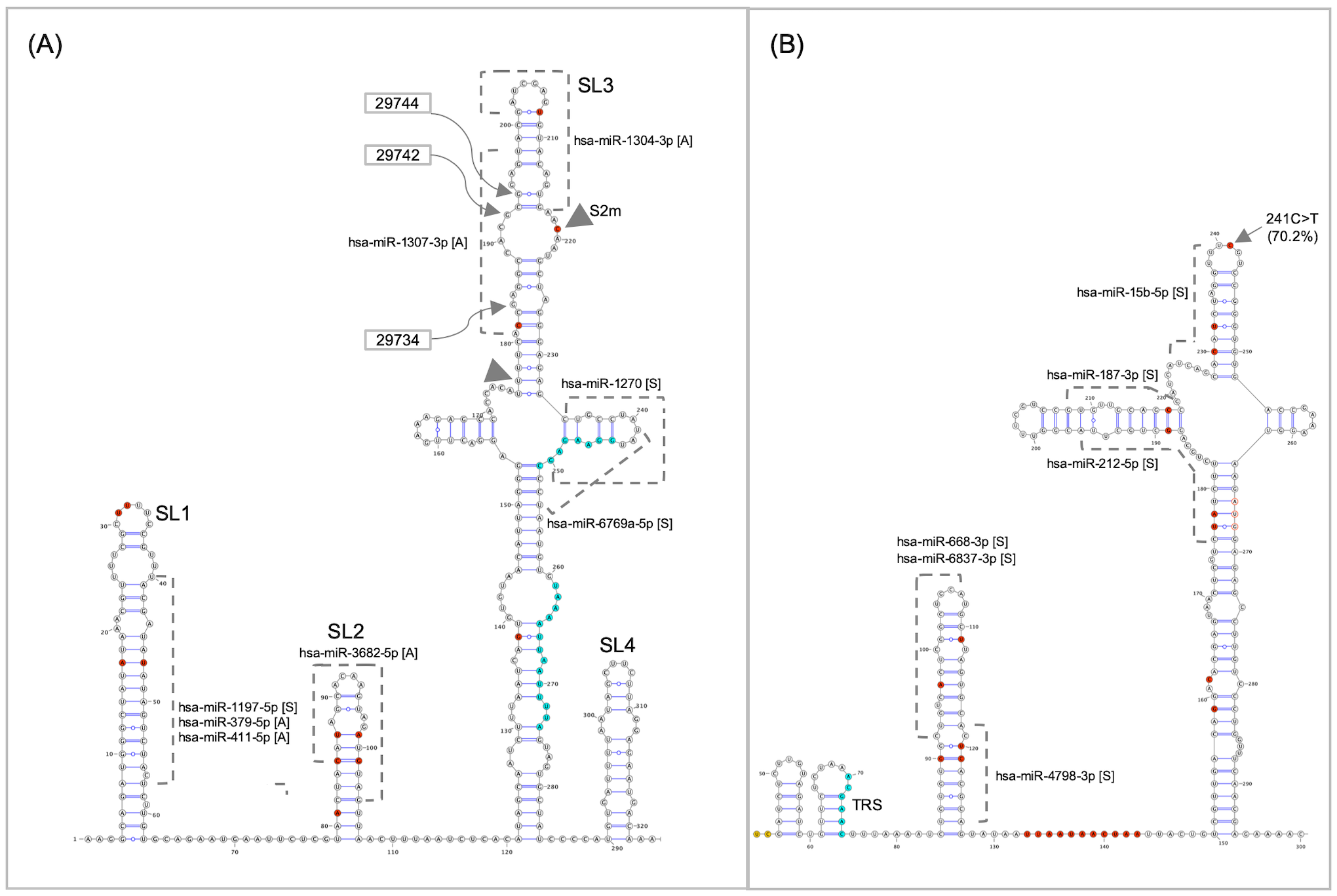

Supplement: FIG S5 [file mSphere.00754-20-sf005.tif]
